# Recurrent Hypoglycemia and Hypercalcemia As Presenting Features of CD30-Positive Peripheral T-cell Lymphoma, Not Otherwise Specified

**DOI:** 10.7759/cureus.117300

**Published:** 2026-10-01

**Authors:** Alireza Izadian Bidgoli, Alberto Gomez Usatorres, Hellen Ailette Gomez Usatorres, Alberto Gomez Veliz, Daina Noriega-Toledo

**Affiliations:** 1 Internal Medicine, American University of the Caribbean School of Medicine, Cupecoy, SXM; 2 Internal Medicine, Jackson Memorial Hospital, Miami, USA; 3 Internal Medicine, Universidad Hispanoamericana, San José, CRI; 4 Flow Cytometry, Jackson Memorial Hospital, Miami, USA

**Keywords:** cd30-positive lymphoma, flow cytometry, hypercalcemia, hypoglycemia, not otherwise specified, peripheral t-cell lymphoma

## Abstract

Peripheral T-cell lymphoma, not otherwise specified (PTCL-NOS), is an aggressive mature T-cell neoplasm that may present with nonspecific systemic and metabolic abnormalities. A 73-year-old man with type 2 diabetes mellitus presented with progressive weakness, altered mental status, recurrent hypoglycemia, and hypercalcemia, accompanied by acute kidney injury, pancytopenia, diffuse lymphadenopathy, and splenomegaly. A complete critical-sample evaluation during hypoglycemia was unavailable, and the mechanism of the parathyroid hormone (PTH)-independent hypercalcemia could not be established because PTH-related peptide and vitamin D metabolite measurements were unavailable.

Core biopsy of a left axillary lymph node demonstrated a mature CD4-positive T-cell lymphoma with strong diffuse CD30 expression, reduced CD7, negative anaplastic lymphoma kinase and Epstein-Barr virus-encoded RNA studies, and a Ki-67 proliferation index exceeding 90%, supporting PTCL-NOS after exclusion of other supported mature T-cell lymphoma entities. Classification remained limited by the absence of an excisional biopsy and additional molecular studies. Prednisone cytoreduction was followed by cyclophosphamide, doxorubicin, and prednisone, with brentuximab vedotin planned but not documented as administered. The course was complicated by profound myelosuppression, *Klebsiella pneumoniae* pneumonia, and neutropenic septic shock. Formal response assessment was not possible because complete staging and adequate follow-up were unavailable.

This case highlights the importance of prompt tissue diagnosis when unexplained metabolic abnormalities coexist with cytopenias and generalized lymphadenopathy. Although hypoglycemia and hypercalcemia occurred in association with PTCL-NOS, the available data do not establish a causal or paraneoplastic relationship.

## Introduction

Peripheral T-cell lymphomas (PTCLs) are a heterogeneous group of mature T-cell neoplasms that often behave aggressively [1,2]. Peripheral T-cell lymphoma, not otherwise specified (PTCL-NOS), is a diagnosis of exclusion requiring integrated assessment of morphology, immunophenotype, clinical findings, and relevant viral studies [1,2]. CD30 expression has therapeutic relevance, although it does not independently establish a PTCL subtype [1-3].

Malignancy-associated hypoglycemia can result from tumor production of insulin-like growth factor 2; markedly increased tumor glucose consumption through aerobic glycolysis has also been documented in lymphoma [4,5]. Lymphoma-associated hypercalcemia may arise from increased calcitriol production, parathyroid hormone (PTH)-related peptide secretion, or bone resorption [6]. The concurrent presentation of hypoglycemia and hypercalcemia is clinically unusual in PTCL-NOS because the two abnormalities have distinct potential mechanisms and each has other possible causes [4-6].

In this patient, the metabolic abnormalities occurred alongside cytopenias and generalized lymphadenopathy, prompting investigation that established PTCL-NOS. Diabetes, renal dysfunction, and critical illness complicated interpretation of the hypoglycemia, and the available testing did not establish a lymphoma-mediated mechanism for either metabolic abnormality. We report this case to highlight the value of prompt tissue diagnosis when concurrent metabolic disturbances accompany signs of systemic malignancy, while emphasizing the need to investigate the cause of each disturbance.

## Case presentation

Clinical presentation

A 73-year-old man with a history of three prior cerebrovascular accidents, type 2 diabetes mellitus not previously requiring insulin, coronary artery disease, hypertension, carotid artery stenosis status post right carotid endarterectomy, and extensive peripheral arterial disease status post left superficial femoral artery atherectomy and angioplasty and bilateral common iliac artery stenting presented with approximately one week of progressive fatigue, generalized weakness, and altered mental status. Emergency medical services documented marked hypoglycemia, for which intravenous dextrose was administered. He was subsequently hospitalized after recurrent hypoglycemia and persistent encephalopathy were accompanied by acute kidney injury, hypercalcemia, pancytopenia, and diffuse lymphadenopathy.

Initial assessment and physical exam

On initial evaluation, the patient appeared chronically ill and was intermittently oriented only to person and place. He was tachycardic and initially hypotensive during episodes of shock. Cardiovascular examination demonstrated a regular rhythm without an appreciable murmur. Respiratory examination revealed diminished breath sounds bilaterally, although no focal wheezing or crackles were consistently documented. His abdomen was soft, nondistended, and nontender without guarding or rebound. Jaundice and scleral icterus developed during the hospitalization. Palpable left axillary and inguinal lymphadenopathy was subsequently documented.

Neurologic assessment revealed encephalopathy without a new focal neurologic deficit. His ability to follow commands fluctuated with his metabolic and respiratory status. During later examinations, he was awake and oriented but remained profoundly weak, with an Eastern Cooperative Oncology Group (ECOG) performance status of 3, inability to ambulate independently, and left foot drop. Progressive dependent and lower extremity edema was also observed. Despite recurrent blood-tinged sputum, he was frequently hemodynamically stable between episodes of critical illness.

Laboratory and diagnostic testing

Initial laboratory evaluation demonstrated decreased serum glucose; increased total and ionized calcium, blood urea nitrogen, and creatinine; decreased estimated glomerular filtration rate; and pancytopenia characterized by leukopenia, anemia, and thrombocytopenia. Additional abnormalities included decreased total protein, albumin, and magnesium; increased hepatic aminotransferases and total bilirubin; and prolonged prothrombin time and international normalized ratio. Renal dysfunction subsequently worsened, with further increases in blood urea nitrogen and creatinine and a decrease in estimated glomerular filtration rate. Hypernatremia and hyperchloremia developed during the period of critical illness and were accompanied by decreased bicarbonate. Direct hyperbilirubinemia and transaminitis also progressed, and lactate dehydrogenase later became markedly elevated (Table 1).

**Table 1:** Summary of laboratory findings at admission and during hospitalization The admission column reflects values obtained at initial presentation. The “peak/nadir during hospitalization” column reports the most clinically significant documented increase or decrease for each parameter during the available hospital course; the direction of the extreme depends on the analyte. The “latest documented value” column reflects the last available measurement in the reviewed record and does not necessarily represent the same hospital day for all analytes. Exact hospital-day assignments could not be reliably reconstructed for all laboratory measurements from the available retrospective documentation. Reference ranges and corresponding units are presented in the final column. A dash indicates that no corresponding result was available

Laboratory parameter	At Admission	Peak/nadir during hospitalization	Latest documented value	Reference range (unit)
Glucose	42	266	144	70-99 mg/dL
Sodium	140	156	140	135-145 mmol/L
Potassium	4.2	3.2	3.9	3.5-5.0 mmol/L
Chloride	104	122	109	98-107 mmol/L
Bicarbonate	22	17	21	22-29 mmol/L
Blood urea nitrogen	27	80	44	7-20 mg/dL
Creatinine	2.60	4.59	1.80	0.70-1.30 mg/dL
Estimated glomerular filtration rate	25	12	39	≥60 mL/minute/1.73 m²
Total calcium	13.1	6.5	6.5	8.6-10.2 mg/dL
Ionized calcium	1.56	0.97	0.97	1.12-1.32 mmol/L
Magnesium	1.2	1.2	1.5	1.7-2.2 mg/dL
Phosphorus	3.4	5.2	3.0	2.5-4.5 mg/dL
Total protein	5.5	4.8	5.0	6.0-8.3 g/dL
Albumin	3.0	2.6	3.0	3.5-5.0 g/dL
Total bilirubin	3.4	7.7	4.3	0.2-1.2 mg/dL
Aspartate aminotransferase	146	172	41	10-40 U/L
Alanine aminotransferase	59	88	54	7-56 U/L
Alkaline phosphatase	111	138	78	44-147 U/L
Lactate dehydrogenase	-	1,170	-	140-280 U/L
White blood cell count	3.3	0.1	0.1	4.0-11.0 × 10³/µL
Absolute neutrophil count	2.6	0.0	0.0	1.5-8.0 × 10³/µL
Hemoglobin	8.9	5.4	7.2	13.5-17.5 g/dL
Hematocrit	29.7	22.3	22.3	41%-53%
Platelet count	67	12	12	150-400 × 10³/µL
Prothrombin time	15.4	18.6	18.2	11.0-13.5 seconds
International normalized ratio	1.21	1.56	1.52	0.8-1.2
Intact parathyroid hormone	-	1.8	-	15-65 pg/mL
Serum monoclonal spike	-	0.00	-	Not detected (g/dL)
Serum free κ:λ ratio	-	0.599	-	0.26-1.65
Plasma Epstein-Barr virus DNA	-	10,400	-	Not detected (IU/mL)
Human T-cell lymphotropic virus I/II DNA	-	Not detected	Not detected	Not detected (qualitative)

The patient initially presented with hypercalcemia, with total calcium of 13.1 mg/dL and ionized calcium of 1.56 mmol/L. During the subsequent hospital course, both total and ionized calcium fell below the reference range, reaching 6.5 mg/dL and 0.97 mmol/L, respectively. The available retrospective record did not permit precise attribution of this transition to a single intervention or mechanism. The change occurred during a complex course involving volume repletion, renal dysfunction, critical illness, and lymphoma-directed therapy; therefore, the subsequent hypocalcemia was interpreted cautiously rather than attributed to a specific treatment effect.

The hypercalcemia was associated with marked suppression of intact PTH. Because the combination of hypercalcemia, renal dysfunction, anemia, and thrombocytopenia initially raised concern for plasma-cell myeloma, serum protein electrophoresis and serum free light-chain testing were obtained. No monoclonal spike was identified, and although both free kappa and lambda light chains were elevated, their ratio remained within the reference range. Cross-sectional imaging showed no destructive or otherwise suspicious osseous lesions, making plasma-cell myeloma less likely. PTH-related peptide, 25-hydroxyvitamin D, and 1,25-dihydroxyvitamin D measurements were not available in the reviewed record; therefore, the specific mechanism of the PTH-independent hypercalcemia could not be established.

The cytopenias were considered multifactorial in the setting of critical illness, inflammation, renal dysfunction, bleeding, and an evolving hematologic malignancy. Haptoglobin was preserved, and the initial ferritin concentration was not markedly elevated. Histologic hemophagocytosis was subsequently identified; however, a complete HLH evaluation, including documented triglyceride, fibrinogen, soluble interleukin-2 receptor, and natural killer-cell functional assessment, was not available. Consequently, the available findings were insufficient to establish lymphoma-associated HLH.

Computed tomography of the chest demonstrated left axillary lymphadenopathy measuring up to 47.5 × 38.2 mm, mild mediastinal and infracarinal lymphadenopathy, a complex 2.7-cm lingular ground-glass and nodular airspace opacity, a 0.6-cm left lower-lobe pulmonary nodule, and bilateral pleural effusions, greater on the right (Figure 1). Computed tomography of the abdomen and pelvis revealed extensive multistation lymphadenopathy involving the pericaval, retroperitoneal, bilateral internal and external iliac, and inguinal nodal stations (Figure 2), together with mild splenomegaly measuring approximately 14.5 cm. Severe chronic right renal atrophy and mild left renal parenchymal scarring were also present. Subsequent staging studies demonstrated suspicious left cervical and prepectoral lymph nodes, persistent thoracic and axillary adenopathy, splenomegaly measuring up to 15.3 cm, and small-to-moderate-volume ascites. No aggressive bone lesion was identified.

**Figure 1:**
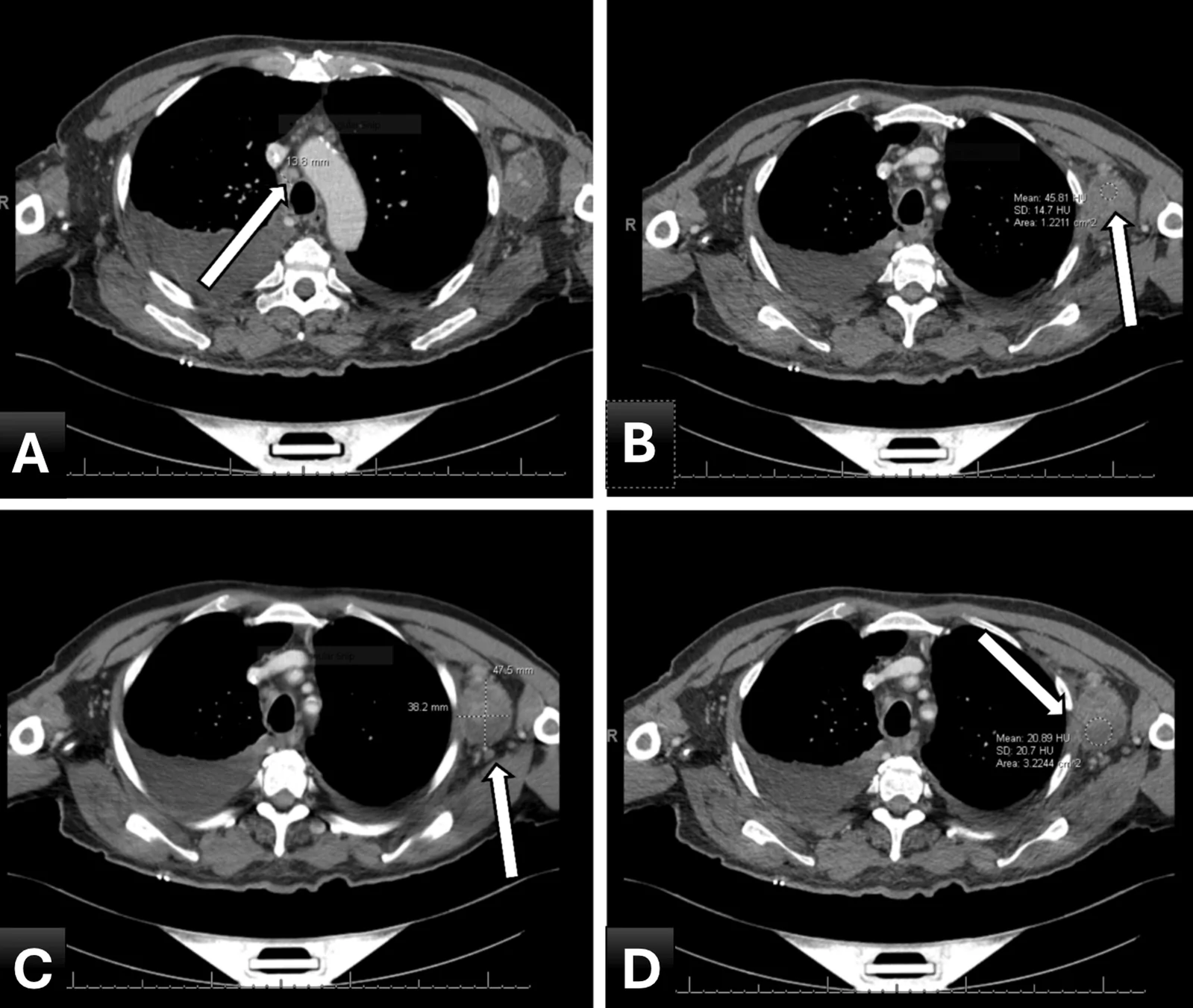
Contrast-enhanced computed tomography of the chest demonstrating mediastinal and left axillary lymphadenopathy Axial contrast-enhanced computed tomography images demonstrate an enlarged mediastinal lymph node measuring approximately 13.8 mm (A, arrow) and multiple enlarged left axillary lymph nodes (B-D, arrows), including a dominant nodal mass measuring approximately 47.5 × 38.2 mm (C). A right-sided pleural effusion is also evident. These findings demonstrated multistation thoracic nodal disease and identified an accessible left axillary lymph node for tissue sampling

**Figure 2:**
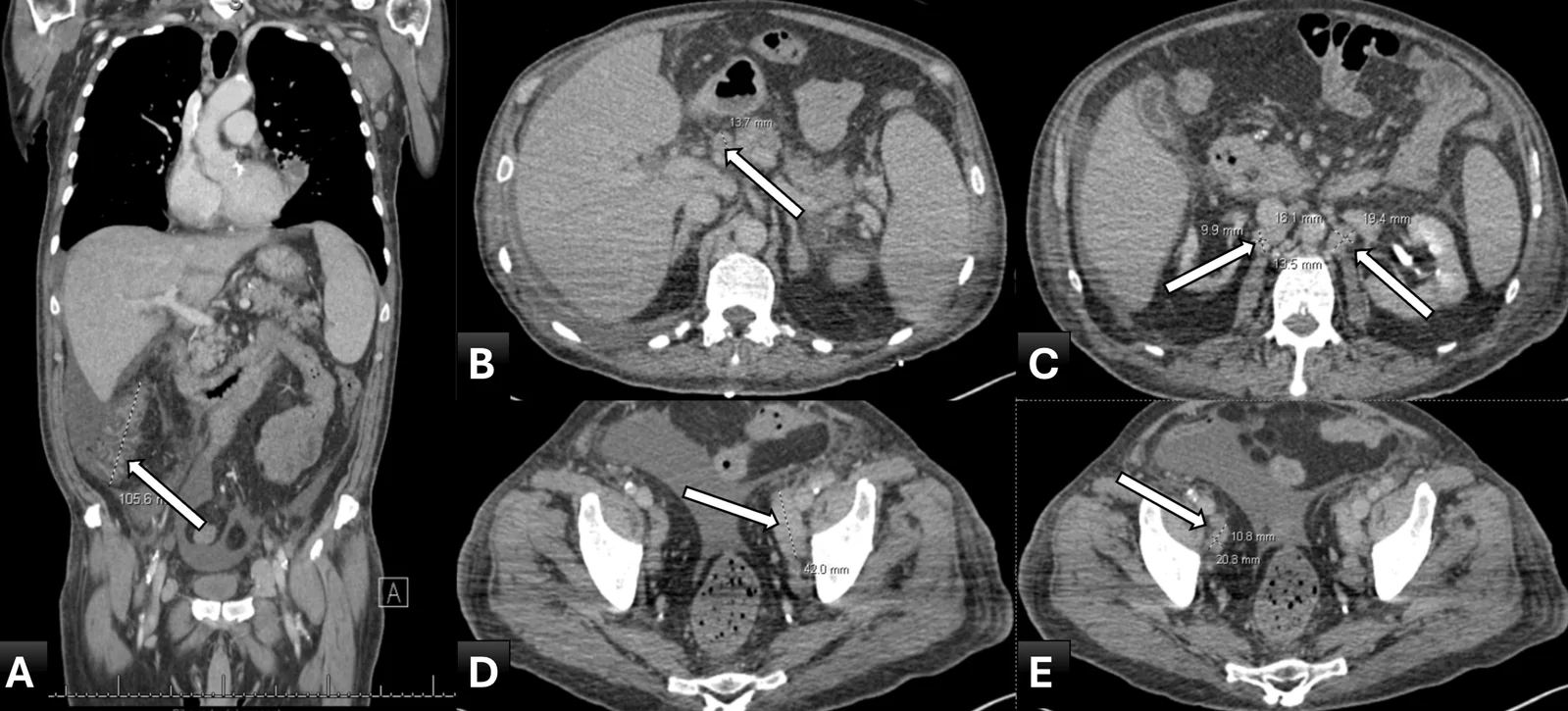
Contrast-enhanced computed tomography demonstrating multistation abdominal and pelvic lymphadenopathy Coronal and axial contrast-enhanced computed tomography images demonstrate extensive multistation abdominal and pelvic lymphadenopathy. (A) Coronal image shows an elongated right pericaval/retroperitoneal nodal conglomerate extending approximately 105.6 mm craniocaudally. (B) Axial image demonstrates an enlarged upper abdominal lymph node measuring approximately 13.7 mm. (C) Multiple aortocaval and para-aortic lymph nodes are present, measuring up to approximately 19.4 mm. (D) A dominant left iliac-chain lymph node measures approximately 42.0 mm. (E) An enlarged right iliac-chain lymph node measures approximately 20.8 × 10.8 mm. Collectively, these findings demonstrate extensive multistation abdominal and pelvic nodal involvement

A bone marrow biopsy was not documented during the acute hospitalization despite the presence of pancytopenia, and formal Ann Arbor staging and International Prognostic Index (IPI)/Prognostic Index for Peripheral T-Cell Lymphoma (PIT) risk scores were not recorded. Lactate dehydrogenase subsequently increased to 1,170 U/L, although a pretreatment baseline value was not available. Uric acid was monitored during tumor-lysis surveillance, but a baseline value was not documented in the available record. Pleural-fluid cultures were negative; however, pleural-fluid cytology, ascitic-fluid cytology, and liver biopsy findings were not available. Prechemotherapy hepatitis B, hepatitis C, and HIV serologies were also not documented in the records available for this report. The absence of these data limited complete retrospective staging and risk stratification.

Core biopsy of a left axillary lymph node showed extensive involvement by a mature T-cell lymphoma composed of large atypical lymphoid cells with frequent mitoses and a Ki-67 proliferation index exceeding 90%. Background histiocytes with hemophagocytosis were present. The neoplastic cells expressed CD3, CD5, CD4, MUM1, B-cell lymphoma 2, and strong diffuse CD30, with decreased CD7, and were negative for anaplastic lymphoma kinase (ALK), granzyme B, T-cell-restricted intracellular antigen-1, T-cell receptor gamma, CD10, B-cell lymphoma 6, PD-1, ICOS, CXCL13, CD34, terminal deoxynucleotidyl transferase, and TCL1. Epstein-Barr virus-encoded RNA (EBER) was negative. Flow cytometry demonstrated an aberrant mature CD4-positive T-cell population comprising approximately 92% of T cells and 53% of all leukocytes, with dim CD3, bright CD5, and variably decreased CD7 expression (Figure 3).

**Figure 3:**
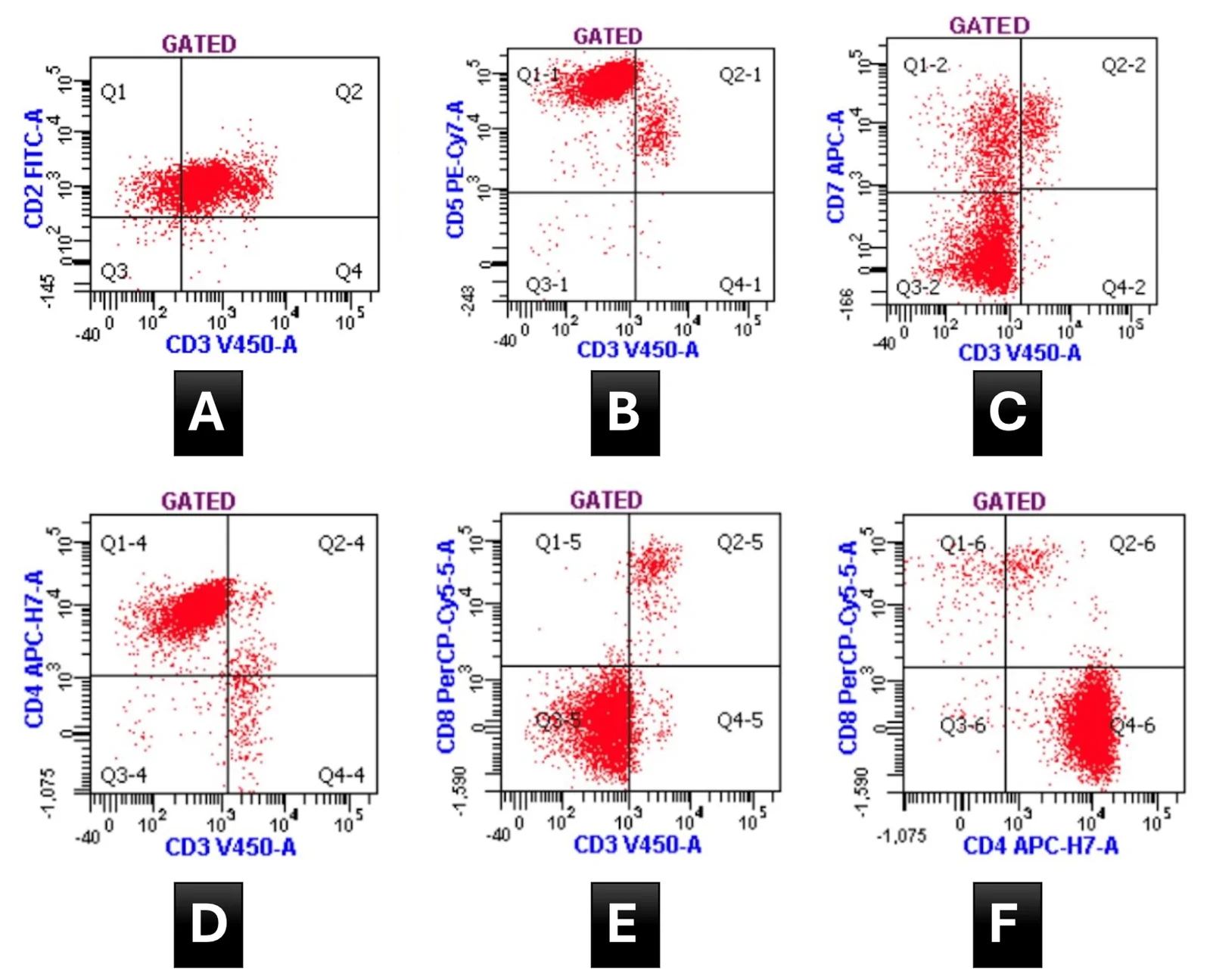
Flow-cytometric immunophenotyping of the left axillary lymph-node specimen Representative dot plots demonstrate an aberrant mature T-cell population with preserved CD2 (A), bright CD5 (B), variably decreased CD7 (C), CD4 expression with dim or partial CD3 (D), and a predominantly CD8-negative (E) and CD4-positive/CD8-negative phenotype (F). These findings, interpreted in conjunction with the morphologic, immunohistochemical, and virologic findings, supported the diagnosis of peripheral T-cell lymphoma, not otherwise specified CD: cluster of differentiation

Strong diffuse CD30 expression raised consideration of ALK-negative anaplastic large-cell lymphoma; however, characteristic hallmark-cell morphology was not identified. ALK negativity excluded ALK-positive anaplastic large-cell lymphoma but did not itself exclude the ALK-negative subtype. No additional molecular studies or excisional biopsy were documented, and assessment of nodal architecture was limited by the core-biopsy specimen. Overall, the available findings supported CD30-positive PTCL-NOS.

Multidisciplinary board discussion

The case was evaluated collaboratively by hematology and oncology, critical care, pulmonology, nephrology, gastroenterology, interventional pulmonology, infectious disease, and pathology. The initial differential diagnosis included plasma-cell myeloma with extramedullary disease, B-cell lymphoma, metastatic malignancy, sarcoidosis, granulomatous infection, adult T-cell leukemia/lymphoma (TCL1), and other mature T-cell lymphoproliferative disorders. The absence of an M-protein, preservation of the serum free light-chain ratio, and lack of destructive bone lesions substantially reduced the likelihood of myeloma. Flow cytometry redirected the evaluation from a presumed B-cell process toward a mature CD4-positive T-cell neoplasm. Pathologic review subsequently established strong diffuse CD30 expression but showed neither ALK expression nor classic anaplastic large-cell morphology. The absence of T-follicular helper markers, cytotoxic markers, T-cell receptor gamma expression, and human T-cell lymphotropic virus (HTLV) infection further narrowed the diagnosis to CD30-positive PTCL-NOS.

The group also considered whether the observed hemophagocytosis represented lymphoma-associated hemophagocytic lymphohistiocytosis (HLH). However, the available clinical and laboratory findings did not establish the full diagnostic criteria for that syndrome, and hemophagocytosis was therefore documented as a pathologic finding rather than treated as a definitive separate diagnosis. Management decisions were further complicated by poor performance status, acute kidney injury, hepatic dysfunction, persistent bleeding, recurrent infection, and severe baseline cytopenias. After stabilization from the initial respiratory and hemodynamic decompensation, the potential benefit of prompt lymphoma-directed therapy was judged to outweigh the substantial treatment-related risks.

Diagnosis and management

The final diagnosis was CD30-positive PTCL-NOS, presenting with diffuse lymphadenopathy, splenomegaly, pancytopenia, hypercalcemia, acute kidney injury, recurrent hypoglycemia, and metabolic encephalopathy. The tumor was ALK-negative, EBER-negative, and HTLV-1/2-negative, with a Ki-67 proliferation index exceeding 90%. Although strong diffuse CD30 expression raised concern for ALK-negative anaplastic large-cell lymphoma, characteristic hallmark-cell morphology was absent. No T-cell receptor clonality testing, DUSP22 or TP63 rearrangement studies, excisional biopsy, or additional expert hematopathology review were documented. Because diagnosis was based on a core biopsy, limited assessment of nodal architecture remained an important diagnostic limitation.

Supportive management included intravenous dextrose and volume repletion for hypoglycemia and hypercalcemia, treatment of acute kidney injury and hypernatremia, broad-spectrum antibiotics and norepinephrine for septic shock, and mechanical ventilation for respiratory failure. Pleural effusion, hemoptysis, melena, and anemia were managed with thoracentesis, conservative measures, proton-pump inhibitor therapy, and transfusion support as clinically indicated.

Prednisone 50 mg twice daily for five days was given for cytoreduction, followed by cyclophosphamide, doxorubicin, and prednisone, with brentuximab vedotin planned as an outpatient component. Tumor-lysis prophylaxis, antimicrobial prophylaxis, growth-factor support, and blood-product transfusions were provided as needed.

Clinical outcome

The initial septic shock and respiratory failure improved, allowing discontinuation of vasopressors, extubation, and transfer from the intensive care unit to the medical floor. Hypercalcemia and hypernatremia resolved with supportive treatment, and renal function improved substantially, although it remained abnormal at the latest assessment. Interval imaging demonstrated stable-to-decreased abdominopelvic lymphadenopathy; however, this occurred after corticosteroid exposure and should not be interpreted as a definitive treatment response. Formal response assessment was not possible because complete staging and adequate post-treatment follow-up were unavailable.

Following the first inpatient chemotherapy cycle, the patient developed profound myelosuppression characterized by marked leukopenia and neutropenia, worsening anemia, and severe thrombocytopenia. He subsequently developed high-grade fever, worsening hypoxemia, encephalopathy, and profound hypotension requiring rapid-response activation and transfer back to the medical intensive care unit. Respiratory culture showed heavy growth of *Klebsiella pneumoniae*, and the clinical presentation was consistent with neutropenic septic shock and pneumonia. He was treated with meropenem and vancomycin, intravenous fluids, albumin, stress-dose hydrocortisone, transfusion support, supplemental oxygen, and norepinephrine. At the time of the latest available documentation, he remained hospitalized in the intensive care unit receiving treatment for neutropenic septic shock and acute hypoxemic respiratory failure; subsequent discharge status, longer term oncologic response, and survival outcome were unavailable. Table 2 summarizes the major clinical events.

**Table 2:** Summary of the patient’s clinical course, diagnostic evaluation, treatment, and major complications Chronological summary of the major clinical events during the hospitalization, including the initial metabolic presentation, diagnostic evaluation, pathologic diagnosis, treatment, and subsequent complications. The sequence reflects the available medical record; exact calendar dates are not shown where they could not be reliably established EMS: emergency medical services; CT: computed tomography; ALK: anaplastic lymphoma kinase; HTLV-1: human T-cell lymphotropic virus 1; HTLV-2: human T-cell lymphotropic virus 2; PTCL-NOS: peripheral T-cell lymphoma, not otherwise specified; WBC: white blood cell; ICU: intensive care unit

Clinical phase	Major events and findings	Management/outcome
Presentation	Approximately one week of progressive fatigue, generalized weakness, and altered mental status. EMS documented glucose of 42 mg/dL	Intravenous dextrose administered before hospital arrival; patient admitted for further evaluation
Initial hospital evaluation	Recurrent hypoglycemia and encephalopathy accompanied by hypercalcemia (13.1 mg/dL), acute kidney injury (creatinine 2.60 mg/dL), pancytopenia, and diffuse lymphadenopathy. Initial hepatic abnormalities were also present	Supportive metabolic management and evaluation for underlying malignancy initiated
Early clinical deterioration	Presumed pneumonia complicated by septic shock and acute hypoxemic respiratory failure	Broad-spectrum antimicrobial therapy, norepinephrine, endotracheal intubation, and mechanical ventilation
Pulmonary evaluation and stabilization	Bronchoscopy demonstrated bloody airway secretions without an obstructing endobronchial lesion. Bilateral pleural effusions were present	Subsequently extubated to noninvasive ventilatory support and later supplemental oxygen; thoracentesis performed, with negative pleural-fluid cultures
Progressive metabolic and organ dysfunction	Renal dysfunction worsened to creatinine approximately 4.6 mg/dL; hypernatremia reached 156 mmol/L. Direct hyperbilirubinemia and transaminitis progressed. Hypoglycemia had already been present before these abnormalities reached their maximal severity	Intravenous fluids, electrolyte management, glucose supplementation, monitoring, and avoidance of nephrotoxic agents. Renal function subsequently improved without dialysis
Malignancy workup	CT demonstrated extensive cervical, axillary, mediastinal, retroperitoneal, iliac, and inguinal lymphadenopathy with splenomegaly. Plasma-cell myeloma was initially considered because of hypercalcemia, kidney injury, anemia, and thrombocytopenia; no monoclonal spike or destructive bone lesions were identified	Hematology/oncology and multidisciplinary diagnostic evaluation pursued; lymph-node biopsy obtained
Pathologic diagnosis	Left axillary lymph-node biopsy demonstrated an aggressive mature T-cell lymphoma with Ki-67 >90%, strong diffuse CD30, CD3/CD4/CD5 expression, decreased CD7, and negative ALK. Flow cytometry showed a dominant abnormal mature CD4-positive T-cell population. HTLV-1/2 testing was negative	Findings supported CD30-positive peripheral T-cell lymphoma, not otherwise specified (PTCL-NOS)
Additional hospital complications	Recurrent hemoptysis, pleural effusions, melena with acute blood-loss anemia, traumatic urethral injury, hypernatremia, hepatic dysfunction, progressive edema, and severe functional decline	Conservative management of hemoptysis because of thrombocytopenia; proton-pump inhibitor and transfusion support for gastrointestinal bleeding; multidisciplinary supportive care
Initial lymphoma treatment	After stabilization from the first episode of septic shock and respiratory failure, lymphoma-directed treatment was initiated	Prednisone 50 mg twice daily for 5 days for cytoreduction, followed by inpatient cyclophosphamide, doxorubicin, and prednisone. Brentuximab vedotin was planned as an outpatient component. Tumor-lysis prophylaxis and surveillance were provided
Early response	Hypercalcemia and hypernatremia resolved; creatinine improved to approximately 1.8-2.0 mg/dL. Interval imaging showed stable-to-decreased abdominopelvic lymphadenopathy after corticosteroid exposure	Continued monitoring and supportive care. Long-term oncologic response could not yet be determined
Postchemotherapy myelosuppression	Profound cytopenias developed after the first inpatient chemotherapy cycle, including WBC approximately 0.1-0.2 × 10³/µL and platelets approximately 12-14 × 10³/µL	Pegfilgrastim administered; subsequent daily granulocyte colony-stimulating factor recommended; red blood cell and platelet transfusion support provided
Recurrent critical illness	Fever to 39.2°C, worsening hypoxemia, encephalopathy, and profound hypotension developed in the setting of profound neutropenia. Respiratory culture showed heavy growth of Klebsiella pneumoniae	Returned to the medical ICU with neutropenic septic shock and pneumonia; treated with meropenem, vancomycin, intravenous fluids, albumin, stress-dose hydrocortisone, transfusions, supplemental oxygen, and norepinephrine
Latest documented status	Patient remained hospitalized in the ICU with neutropenic septic shock and acute hypoxemic respiratory failure	Subsequent discharge status, long-term lymphoma response, and survival outcome were not available in the reviewed records

## Discussion

Background

PTCL-NOS is an aggressive mature T-cell lymphoma category used when a neoplasm does not fulfill criteria for a more specific T-cell or natural killer (T/NK)-cell entity. Historically, it functioned as a residual category; successive classification advances have narrowed it by recognizing distinct nodal T-follicular helper lymphomas, Epstein-Barr virus (EBV)-associated nodal T/NK-cell lymphomas, and other biologically defined subtypes [7,8].

Mature T-cell lymphomas constitute approximately 10%-12% of lymphoid malignancies, and PTCL-NOS accounts for roughly one-quarter of these tumors in many contemporary series [9]. Incidence and subtype distribution vary substantially by geographic region; population-based data suggest a male predominance and identify PTCL-NOS as one of the most frequent mature T-cell lymphoma subtypes [10]. PTCL-NOS primarily affects adults, often presents later in life, and generally behaves as a systemic malignancy with nodal and extranodal involvement [2,9].

No single hereditary, environmental, or occupational exposure defines PTCL-NOS. Rather, the diagnostic process requires exclusion of entities with distinctive viral, immunophenotypic, or molecular associations, including HTLV-1-associated adult TCL1, EBV-associated nodal T/NK-cell lymphoma, anaplastic large-cell lymphoma, and T-follicular helper lymphomas [7]. Clinically, the expression of CD30 is particularly important because it provides a therapeutically actionable biomarker in systemic PTCL [2].

Pathology/pathophysiology

PTCL-NOS arises from post-thymic mature T cells and is biologically heterogeneous rather than a single molecularly uniform disease. Current studies support molecularly informed subgrouping, including TBX21- and GATA3-associated phenotypes, although these subgroups are not interchangeable with distinct clinicopathologic entities [7]. Immunohistochemical algorithms can reproduce these biologic subgroups, with GATA3-associated tumors tending to demonstrate less favorable outcomes [11]. Recent genomic work has linked the PTCL-GATA3 subtype to cooperating TP53 and PTEN loss and dysregulated T-cell receptor and Janus Kinases/Signal Transducers and Activators of Transcription signaling; these alterations were not evaluated in the present patient and therefore cannot be inferred in this case [12].

The diagnostic hallmark remains integration of morphology, broad T-cell immunophenotyping, flow cytometry, viral studies, and exclusion of better defined T-cell lymphoma entities [7]. In this patient, the left axillary lymph-node core biopsy demonstrated a mature CD4-positive, CD30-strong/diffuse T-cell lymphoma with reduced CD7 expression, absent T-follicular helper markers, negative ALK and EBER studies, and no evidence of HTLV-1 infection, supporting PTCL-NOS. Background hemophagocytosis reflected immune dysregulation within the involved tissue but, in isolation, did not establish HLH. The recurrent hypoglycemia and hypercalcemia may have reflected multifactorial critical illness. Because confirmatory biochemical studies were unavailable, a causal or paraneoplastic relationship between PTCL-NOS and these metabolic abnormalities could not be established.

Literature review

Clinical Presentation

PTCL-NOS commonly presents with advanced-stage nodal disease, constitutional symptoms, extranodal involvement, cytopenias, and laboratory abnormalities related to systemic inflammation or marrow involvement [2,9]. The patient’s age, diffuse lymphadenopathy, splenomegaly, pleural effusions, and cytopenias are therefore consistent with the typical aggressive systemic phenotype. However, his presentation was unusual because recurrent severe hypoglycemia and hypercalcemia were prominent at admission, preceding definitive lymphoma diagnosis. These metabolic disturbances created a diagnostic dilemma because each may also arise from diabetes treatment, reduced nutritional intake, renal dysfunction, critical illness, medications, or infection. The available record does not permit attribution of either abnormality solely to PTCL-NOS.

Diagnostic Workup

Contemporary guidelines emphasize obtaining adequate tissue and using morphology, immunohistochemistry, flow cytometry, EBV testing, and selective viral studies to classify nodal mature T-cell lymphomas accurately [2,7]. This approach was particularly important in the present case because CD30 expression can occur in several lymphoma subtypes and is not by itself diagnostic of PTCL-NOS. The absence of ALK expression, T-follicular helper markers, EBER positivity, and HTLV-1 infection, together with demonstration of an abnormal mature CD4-positive T-cell population on flow cytometry, supported PTCL-NOS after exclusion of competing entities. The background hemophagocytosis further heightened concern for immune dysregulation but required clinical correlation rather than diagnostic overinterpretation.

Cross-sectional imaging in this patient demonstrated multistation lymphadenopathy involving axillary, mediastinal, abdominopelvic, and inguinal stations, with splenomegaly. This distribution is consistent with the disseminated disease frequently observed in PTCL-NOS [9]. Brain magnetic resonance imaging showed no leptomeningeal disease, which was clinically reassuring given the patient’s prior encephalopathy and the need to distinguish central nervous system involvement from metabolic and critical illness-related causes.

Management

For previously untreated CD30-positive systemic PTCL, brentuximab vedotin plus cyclophosphamide, doxorubicin, and prednisone (A+CHP) is a guideline-supported first-line regimen [2]. In the ECHELON-2 trial, A+CHP improved five-year progression-free and overall survival compared with cyclophosphamide, doxorubicin, vincristine, and prednisolone in CD30-positive PTCL [3]. In this patient, ECOG performance status 3, multiorgan dysfunction, severe cytopenias, and active infection complicated treatment selection and timing. After clinical stabilization, prednisone was used as cytoreductive prephase therapy, followed by inpatient cyclophosphamide, doxorubicin, and prednisone. Brentuximab vedotin was planned as an outpatient component but was not documented as administered during the hospitalization; therefore, the patient did not receive a complete A+CHP regimen during the available follow-up. Tumor-lysis monitoring, allopurinol, hydration, growth-factor support, and antimicrobial prophylaxis accompanied treatment initiation.

Outcome and follow-up

The available follow-up was limited by severe intercurrent complications. Although the patient had begun treatment for CD30-positive PTCL-NOS, his course was complicated by chemotherapy-associated profound neutropenia, recurrent septic shock, and respiratory infection with heavy growth of *K. pneumoniae*. Thus, durable response, remission status, and long-term survival cannot be assessed from the available record. This limitation is clinically important because PTCL-NOS remains associated with inferior outcomes compared with many B-cell lymphomas, particularly among older patients with substantial comorbidity [9]. A recent single-center PTCL-NOS series likewise demonstrated modest three-year progression-free survival despite contemporary treatment approaches [13].

Literature Summary

Existing literature emphasizes that PTCL-NOS is a diagnosis of exclusion requiring careful distinction from newly defined nodal T-cell lymphoma entities and that CD30 expression has direct therapeutic relevance [2,7]. This case adds a clinically relevant presentation in which recurrent hypoglycemia and hypercalcemia prompted evaluation that uncovered disseminated CD30-positive PTCL-NOS. It also illustrates that metabolic abnormalities in a medically complex patient should not be assumed to be paraneoplastic without appropriate confirmatory testing, while persistent unexplained cytopenias, hypercalcemia, and generalized lymphadenopathy should prompt expedited tissue diagnosis and multidisciplinary hematopathology review. A summary of the comparative analysis of this case with the current literature is provided in Table 3.

**Table 3:** Comparison of the present case with established features of peripheral T-cell lymphoma, not otherwise specified PTCL-NOS: peripheral T-cell lymphoma, not otherwise specified; ALK: anaplastic lymphoma kinase; EBER: Epstein-Barr virus-encoded RNA; HTLV-1: human T-cell lymphotropic virus 1; HTLV-2: human T-cell lymphotropic virus 2; EBV: Epstein-Barr virus; A+CHP: brentuximab vedotin plus cyclophosphamide, doxorubicin, and prednisone; CHOP: cyclophosphamide, doxorubicin, vincristine, and prednisolone

Domain	Findings reported in the literature	Findings in the present case	Comparison and clinical significance
Patient population	PTCL-NOS predominantly affects adults, frequently older individuals, with a reported male predominance [9,10]	A 73-year-old man with extensive cardiovascular comorbidity	The patient’s age and sex were consistent with the typical demographic profile
Clinical presentation	Patients commonly present with constitutional symptoms, advanced nodal disease, extranodal involvement, splenomegaly, cytopenias, and systemic inflammation [2,9]	Fatigue, weakness, encephalopathy, pancytopenia, diffuse lymphadenopathy, splenomegaly, and pleural effusions	Most clinical and radiographic findings reflected the recognized aggressive systemic phenotype of PTCL-NOS
Metabolic abnormalities	Recurrent hypoglycemia and hypercalcemia are not defining or routinely emphasized presenting features of PTCL-NOS [2,9]	Recurrent severe hypoglycemia and parathyroid hormone-suppressed hypercalcemia were prominent at presentation	The paired metabolic abnormalities were unusual and broadened the differential diagnosis; however, a direct lymphoma-mediated mechanism could not be established.
Disease distribution	PTCL-NOS frequently presents with disseminated nodal and extranodal disease [2,9]	Axillary, mediastinal, cervical, prepectoral, retroperitoneal, pericaval, iliac, and inguinal lymphadenopathy with splenic involvement	The multistation distribution above and below the diaphragm was consistent with advanced systemic disease
Diagnostic approach	Diagnosis requires adequate tissue sampling and integration of morphology, immunohistochemistry, flow cytometry, and viral studies to exclude better-defined T-cell lymphoma entities [1,7,8]	Core biopsy showed a mature CD4-positive, strongly CD30-positive T-cell lymphoma with reduced CD7, dim CD3, and a Ki-67 index exceeding 90%. ALK, EBER, HTLV-1/2, cytotoxic markers, and T-follicular helper markers were negative	The integrated findings supported PTCL-NOS after excluding anaplastic large-cell lymphoma, nodal T-follicular helper-cell lymphoma, adult T-cell leukemia/lymphoma, and EBV-associated disease
Molecular characterization	GATA3- and TBX21-associated molecular subgroups have been described and may carry prognostic significance [2,7,11]	Comprehensive genomic profiling and molecular subgroup classification were unavailable	The absence of molecular testing limited further biologic and prognostic characterization but did not prevent clinicopathologic diagnosis
Management	A+CHP is a guideline-supported first-line regimen for eligible patients with CD30-positive systemic PTCL and has demonstrated improved survival outcomes compared with CHOP [2,3]	Prednisone cytoreduction was followed by inpatient cyclophosphamide, doxorubicin, and prednisone; brentuximab vedotin was planned but was not documented as administered during the available follow-up	The intended approach was consistent with an A+CHP-based strategy, although critical illness prevented confirmation of complete regimen delivery
Outcome and follow-up	PTCL-NOS is associated with unfavorable outcomes, particularly in older patients with advanced disease and substantial comorbidity [9,13]	Treatment was complicated by profound myelosuppression, Klebsiella pneumoniae pneumonia, neutropenic septic shock, and recurrent respiratory failure. Long-term response and survival were unavailable	The clinical course illustrates the substantial treatment-related risks in medically fragile patients, but limited follow-up prevented assessment of remission or long-term prognosis

What we learned from this case

This case demonstrates that recurrent unexplained hypoglycemia, particularly in a patient not receiving chronic insulin therapy, and PTH-independent hypercalcemia warrant investigation beyond common metabolic and medication-related causes. The concurrent presence of pancytopenia, acute kidney injury, splenomegaly, and multistation lymphadenopathy represented a particularly important signal of an underlying systemic malignancy. Moreover, the absence of an M-protein and a normal serum free light-chain ratio illustrate that an unrevealing initial myeloma evaluation should not prematurely end the hematologic workup.

Cross-sectional imaging and timely biopsy of an accessible lymph node were essential for establishing the diagnosis. Accurate classification required integration of histologic morphology, immunohistochemistry, flow cytometry, and virologic testing because PTCL-NOS is fundamentally a diagnosis of exclusion. Clinicians should therefore prioritize tissue acquisition when unexplained metabolic abnormalities coexist with cytopenias or generalized lymphadenopathy.

The clinical course also emphasizes the competing risks encountered during treatment of aggressive lymphoma. Metabolic stabilization and lymphoma-directed therapy must be accompanied by tumor lysis surveillance, renal and electrolyte monitoring, antimicrobial prophylaxis, growth-factor support, and rapid evaluation of fever or hemodynamic deterioration. Although this case cannot establish that the hypoglycemia and hypercalcemia were directly caused by the lymphoma, it broadens the diagnostic framework for these abnormalities and suggests that their recurrence may justify close metabolic surveillance during staging and treatment.

Limitations

This report is limited by its retrospective nature and reliance on documentation from a prolonged, clinically complex hospitalization. A time-matched critical-sample evaluation during spontaneous hypoglycemia was not available; specifically, insulin, C-peptide, β-hydroxybutyrate, cortisol, and sulfonylurea screening were not documented in the reviewed record. The outpatient antihyperglycemic regimen, timing of the last doses, preadmission nutritional intake, and exact frequency and timing of recurrent hypoglycemic episodes were also incompletely documented. Consequently, contributions from medications, reduced intake, renal or hepatic dysfunction, and critical illness could not be distinguished from a potential malignancy-associated mechanism. The hypercalcemia was likewise incompletely characterized because PTH-related peptide, 25-hydroxyvitamin D, and 1,25-dihydroxyvitamin D results were unavailable.

Additional limitations affected lymphoma classification, staging, and outcome assessment. Diagnosis was based on a core lymph-node biopsy without an excisional specimen or additional molecular studies, limiting evaluation of nodal architecture and further exclusion of competing mature T-cell lymphoma entities. A bone marrow biopsy, formal Ann Arbor stage, IPI/PIT risk scores, complete HLH evaluation, and several baseline staging studies were not documented. Severe infection, multiorgan dysfunction, and treatment-associated cytopenias further complicated interpretation of the clinical course. Complete staging, formal treatment-response assessment, postdischarge follow-up, and long-term survival data were unavailable. Accordingly, this single case should be considered hypothesis-generating and does not establish a causal relationship between PTCL-NOS and either hypoglycemia or hypercalcemia.

## Conclusions

This case describes a 73-year-old man with recurrent hypoglycemia, PTH-independent hypercalcemia, pancytopenia, acute kidney injury, and generalized lymphadenopathy who was ultimately diagnosed with CD30-positive PTCL-NOS. Although the metabolic abnormalities occurred in association with the lymphoma, the available data were insufficient to establish a causal or paraneoplastic relationship, and their etiology was likely multifactorial. Unexplained hypoglycemia and PTH-independent hypercalcemia occurring alongside cytopenias and generalized lymphadenopathy should prompt evaluation for an underlying hematologic malignancy, with early tissue acquisition and integrated pathologic assessment remaining essential for diagnosis.
